# Supplementary Light Source Affects the Growth and Development of *Codonopsis lanceolata* Seedlings

**DOI:** 10.3390/ijms19103074

**Published:** 2018-10-08

**Authors:** Xiuxia Ren, Ya Liu, Hai Kyoung Jeong, Byoung Ryong Jeong

**Affiliations:** 1 Division of Applied Life Science (BK21 Plus Program), Graduate School, Gyeongsang National University, Jinju 52828, Korea; xiuxia0823@163.com (X.R.); liuya113@mails.ucas.ac.cn (Y.L.); hksmile@naver.com (H.K.J.); 2 Institute of Agriculture and Life Science, Gyeongsang National University, Jinju 52828, Korea; 3 Research Institute of Life Science, Gyeongsang National University, Jinju 52828, Korea

**Keywords:** supplementary light, medicinal plant, physiology, propagation, stress, western blot

## Abstract

*Codonopsis lanceolata* is widely used in traditional medicine and diets. However, there is no optimal protocol for the commercial production of *C. lanceolata* seedlings. This study was carried out to find the optimum supplementary light source for the production of *C. lanceolata* seedlings. Seedlings were grown for four weeks in a glasshouse with an average daily light intensity of 490 μmol·m^−2^·s^−1^ photosynthetic photon flux density (PPFD) coming from the sun and a 16-h daily supplementary lighting at 120 μmol·m^−2^·s^−1^ PPFD from either high-pressure sodium (HPS), metal halide (MH), far-red (FR), white LED (LED-w), or mixed (white: red: blue = 1:2:1) LEDs (LED-mix). The results showed that the greatest total biomass, stem diameter, ratio of shoot weight to shoot length, root biomass, and ratio of root weight to shoot weight were found in seedlings grown under supplementary LED-mix. Meanwhile, the stomatal properties and soluble sugar contents were improved for seedlings in LED-mix. The contents of starch, total phenols, and flavonoids were the greatest for seedlings in LED-w and LED-mix. The expression of photosynthetic proteins and genes in seedlings was also enhanced by LED-mix. Overall, these results suggest that LED-mix is advantageous to the photosynthetic potential and the accumulation of biomass, carbohydrates and secondary metabolites in *C. lanceolata*.

## 1. Introduction

*Codonopsis lanceolata*, commonly known as the bonnet bellflower, belongs to the Campanulaceae family. It is a perennial herb widely distributed in Asia [1]. The roots of *C. lanceolata* are highly valued in traditional Chinese medicine, and are consumed as a high-class vegetable in certain Asian countries, particularly in Republic of Korea [2]. Biologically active compounds, including saponins, polyphenols, triterpene, tannins, alkaloids, and steroids are abundant in *C. lanceolata*, contributing to its numerous medicinal properties [3]. The claimed therapeutic values of *C. lanceolata* include treatments for coughs, bronchitis, obesity, cancer, colitis, and hepatitis [3]. In recent years, the demand for *C. lanceolata* roots has highly accelerated. However, traditional extensive cultivation with poor seedling management have hampered its large-scale cultivation. Hence, an optimal protocol for the commercial production of *C. lanceolata* is greatly needed.

Light is important in regulating the photosynthesis and photomorphogenesis of plants, particularly for those grown in controlled environments [4]. Changes in the light spectrum evoke different morphology, physiology, biochemistry, and transcriptional expression in plants [5,6,7]. The leaf structure, thicknesses of the palisade mesophyll cells, spongy tissues, and epidermis vary with the light spectrum [8,9]. Additionally, the development of the stomata and its properties can be largely affected by the light environment [10,11]. Light quality alters photosynthesis by changing the photosynthetic apparatus activity and the expression and/or the activity of photosynthetic enzymes [6]. It has been reported that the chlorophyll level, chloroplast number, and abundance of chloroplast/thylakoid proteins, including light-harvesting complexes (LHC), cytochrome b6f (Cyt b6f), photosystem I (PSI), photosystem II (PSII), and ATP synthase (ATPase), could change under long-term light treatments [12].

Plants mediate a wide range of spectrum-dependent responses through unique photoreceptors including cryptochromes, phototropins, and phytochromes [8]. Cryptochromes and phototropins are sensitive to blue light, whereas phytochromes are sensitive to red light. Blue and red lights induce maximum photosynthesis, since they are absorbed more efficiently by the photosynthetic pigments compared to lights of other wavelengths. Red and blue lights largely affect the photomorphogenesis, development and activity of the photosynthetic apparatus, chloroplast movement, and stomatal opening [10]. The combination of red and blue lights allows for a more accelerated photosynthesis than either red or blue light alone [13]. Far-red (FR) light, at the extreme red end of the visible spectrum, leads to increases in the stem elongation, hypocotyl elongation, leaf area, and growth rate of plants [14,15]. White light is believed to produce regular leaf morphology and a high photosynthetic rate [16]. Such photo-responses are of practical importance in plant cultivation technologies, since the feasibility of tailoring illumination spectra purposefully enables one to control plant growth and development. In this case, a mixture of red, blue, and white lights was the most effective for high quality seedling production [17,18,19,20,21].

Based on light source, horticultural cultivation facilities can be separated into two main categories: greenhouses and entirely enclosed plant factories [22]. Supplementary lighting can provide the necessary light for plant growth when natural light is insufficient in a greenhouse, particularly during rainy seasons. Improved light conditions in greenhouses can optimize plant growth and the accumulation of phytochemical substances [23]. Conventional artificial light sources usually used in cultivation facilities include high pressure sodium (HPS), metal halide (MH), incandescent, and fluorescent lamps [22] that provide a wide range of wavelengths. HPS and MH are usually used as commercial supplementary lights in greenhouses due to their low cost, long lifetime, and reasonable light spectra [24]. However, there are certain disadvantages associated with these light sources in the cultivation of vegetables or medicinal plants. Light-emitting diodes (LEDs) have some unique advantages compared to these conventional artificial light sources, including low weight and volume, long life, wavelength specificity, low energy consumption, minimal heating, and high energy efficiency [25,26].

To improve growth and accelerate the commercial production of *C. lanceolata* seedlings, different supplementary light sources, including HPS, MH, white LED (LED-w), and a mixture of red, blue and white LED (LED-mix) were used during a rainy summer season. Moreover, to investigate how the light environment drives plant growth and photosynthesis, the stomatal properties, accumulation of primary and secondary metabolites, and expression of photosynthetic and energy-related genes and proteins were monitored.

## 2. Results

### 2.1. Morphology and Growth Parameters Analysis

**Figure 1:**
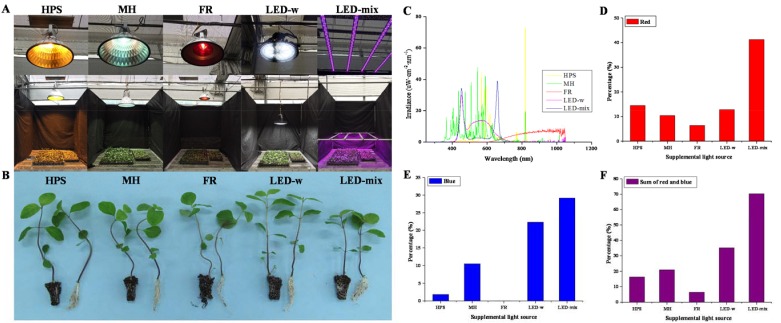
Supplementary light sources (**A**), the morphology of *Codonopsis lanceolata* seedlings affected by the supplementary light sources (**B**), spectral analysis of the high-pressure sodium (HPS, plotted in yellow), metal halide (MH, plotted in green), Far-red light (FR, plotted in red), white light emitting diode (LED-w, plotted in rosy red), and a mixture of red, blue, and white LED (LED-mix, plotted in blue) each at a total irradiance of 3000 μW·cm^−2^ (**C**), and the percentage of red and/or blue light irradiance (**D**–**F**).

An HPS lamp produced a characteristic golden-orange light (Figure 1A). Similar with the spectral identification in a previous report [27], the strongest wavelength emitted by HPS was 819–821 nm (FR), followed by 570 and 583–617 nm (yellow and orange) (Figure 1C). The largest accumulated irradiance of HPS appeared in the orange range, followed by FR (Table 1). Blue and red lights in HPS took up about 16.3% of the total irradiance. MH was white in appearance. Strong emission lines of MH were largely dispersed in all ranges. About 50% of the total MH irradiance was focused on green and violet lights, with the rest of the irradiance distributed in other wavelengths. Blue and red lights accounted for about 20.9% of the total MH irradiance. FR light appeared as dark red, with strong emission lines found at 620–1050 nm (red and far-red). FR and IR took up 93.3% of FR light’s total irradiance, while red and orange lights occupied about 6.5%. Light of LED-w appeared white. LED-w had a wide range of irradiance from 430 to 670 nm (from violet to red). Interestingly, the peak of LED-w irradiance was green light, followed by blue light. Red and blue lights accounted for 35.1% of the total LED-w irradiance. LED-mix consisted of a mixture of red, blue and white lights with peak emission at 435–480 nm (blue) and 634–670 nm (red). Furthermore, the accumulated irradiance of LED-mix was mainly centered on red and blue lights, which accounted for 70.3% of the total irradiance. Overall, the largest proportions of red light, blue light, and the sum of these two were the highest in LED-mix, followed by LED-w, and were the lowest in FR (Figure 1D–F). The summed proportion of blue and red lights was quite consistent with the blue light proportion. However, the red light proportion had a different pattern than that of the blue light proportion, since HPS contained a relatively higher red light proportion compared to MH.

**Figure 2:**
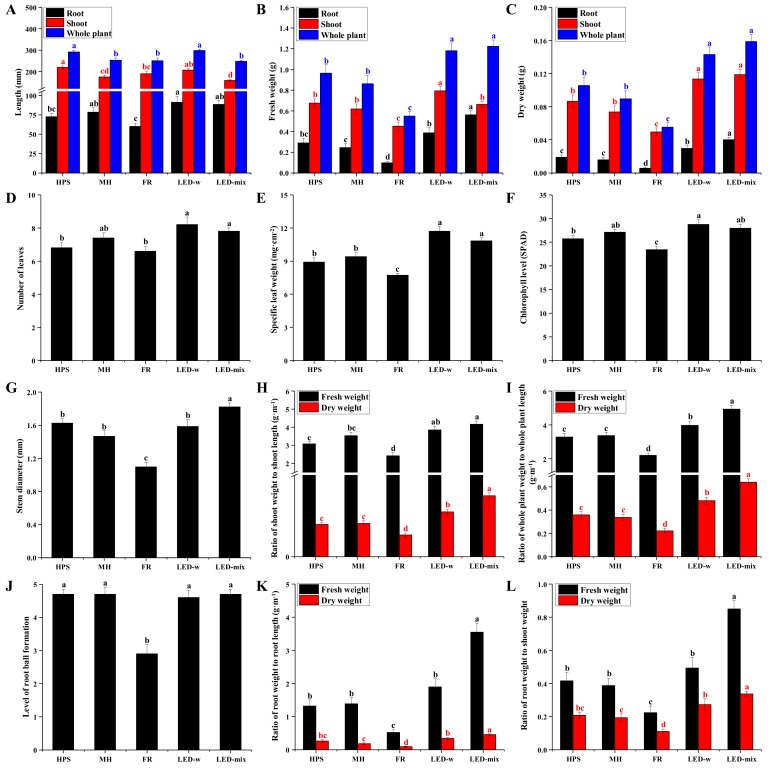
The growth parameters of *C. lanceolata* seedlings as affected by supplementary light sources: (**A)**, Length of root, shoot and whole plant; (**B**), Fresh weights of root, shoot and whole plant; (**C**), Dry weights of root, shoot and whole plant; (**D**), Number of leaves; (**E**), Specific leaf weight; (**F**), Chlorophyll level; (**G**), Stem diameter; (**H**), Ratio of shoot weight to shoot length; (**I**), Ratio of whole plant weight to whole plant length; (**J**), Level of root ball formation; (**K**), Ratio of root weight to root length; and (**L**), Ratio of root weight to shoot weight. Different small letters indicate the significant separation within treatments by Duncan’s multiple range test at a 0.05 level.

After four weeks of cultivation, different morphologies were observed in *C. lanceolata* seedlings (Figure 1B). A long, thin, and vine-like stem was characteristic of seedlings grown in FR, while a stocky and upright stem was observed in seedlings grown under LED-mix. Growth parameters (Figure 2) of *C. lanceolata* seedlings were in accordance with the morphology. Shoot length was the smallest for seedlings in LED-mix. Root length was higher for seedlings in LED-w (+25.8%, +16.2%) and LED-mix (+22.1%, +12.7%), and lower for seedlings in FR (−13.2%, −8.9%) compared to that of seedlings in HPS and MH. Total fresh and dry weights of seedlings were dramatically enhanced by LED-w and LED-mix, and significantly decreased by FR, compared to those of seedlings grown in HPS and MH. The changes in the dry weight was obviously larger. The number of leaves, specific leaf weight, and chlorophyll level were significantly higher for seedlings grown in LED-w and LED-mix, whereas they were largely decreased for seedlings grown in FR. Moreover, the total biomass, stem diameter, and compactness properties of shoots and roots were the most increased for seedlings grown in LED-mix, followed by seedlings in LED-w, and were the lowest for seedlings in FR. The ratio of total dry weight to whole plant length, shoot dry weight to shoot length, root dry weight to root length, and root fresh weight to shoot fresh weight for seedlings in LED-mix were about twice that of seedlings in HPS and MH. The ratio of root fresh weight to root length was more than 2.5-fold higher for seedlings in LED-mix compared to that of seedlings in HPS and MH. The level of root ball formation for seedlings in FR was significantly lower than that of seedlings in the other four light environments.

### 2.2. Stomatal Properties Analysis and Micrographs

**Figure 3:**
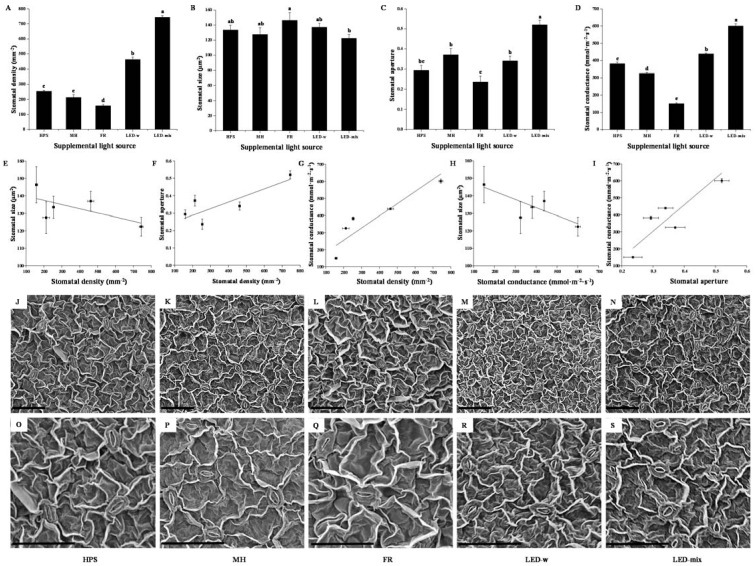
Stomatal density (**A**), stomatal size (**B**), stomatal aperture (**C**), stomatal conductance (**D**), relationships between the stomatal properties (**E**–**I**), and stomatal micrographs (**J**–**S**) of *C. lanceolata* seedling leaves observed by a scanning electron microscope. Scale bar, 50 µm. Different small letters indicate the significant separation within treatments by Duncan’s multiple range test at a 0.05 level.

The supplementary light sources exerted a considerable influence on the stomatal properties of *C. lanceolata* leaves during their development (Table 2 and Figure 3). Several basic characteristics were displayed in Table 2. Guard cells and stomata pores were shorter for *C. lanceolata* grown under FR, LED-w, and LED-mix than those of seedlings in HPS and MH. Seedlings grown in FR had a greater width of the guard cell pair than that of seedlings in the other four treatments. Compared to seedlings in HPS or MH, the stomata pore width of leaves was smaller for seedlings in FR and LED-w, and greater for seedlings in LED-mix.

Further stomatal properties and micrographs were shown in Figure 3. LED-mix was the most effective in enhancing the stomatal density in seedlings, where it was 2.9-time higher than that of seedlings in HPS and 3.5-times higher than that of seedlings in MH (Figure 3A). LED-w was the second most effective in enhancing, whereas FR reduced, the stomatal density. Supplementary lighting affected the stomatal aperture similarly as it did the stomatal density (Figure 3C,F). Interestingly, the stomatal size in *C. lanceolata* leaves presented an opposite trend in response to supplementary light compared to the behavior of stomatal density (Figure 3B,E). The stomatal size was the greatest in leaves developed in FR and the least in leaves grown under LED-mix. A correlational analysis showed that the stomatal size is negatively correlated with the stomatal conductance (Figure 3H), while the stomatal density and aperture were positively correlated with the stomatal conductance (Figure 3G,I). Stomatal micrographs certified the results presented above. FR produced leaves with a lower number of larger stomata, while LED-mix and LED-w produced leaves with a higher number of smaller stomata (Figure 3J–S).

### 2.3. Primary and Secondary Metabolites Analysis

The contents of primary and secondary metabolites were highly affected by the supplementary light sources (Table 3). Soluble sugar content was significantly higher for seedlings in LED-mix (+23.1% and +30.5%) and lower for those in FR (−36.4% and −32.6%) and in LED-w (−42.5% and −39.0%) compared with that of seedlings in HPS and MH. The highest starch content was found in seedlings grown under LED-mix, followed by those in LED-w, and was the lowest for seedlings in FR. The protein content was significantly lower for seedlings in HPS than for seedlings in the other four treatments. The accumulation of total phenol and flavonoid was significantly enhanced by LED-w and LED-mix, with levels about twice of those in HPS-grown seedlings and more than 3-times the levels in MH-grown seedlings. Seedlings grown in FR had significantly lower total phenol and flavonoid contents.

### 2.4. Chloroplast Protein Profiles and Immunoblot Analysis of D_1_ Protein (Anti-PsbA, PSII)

**Figure 4:**
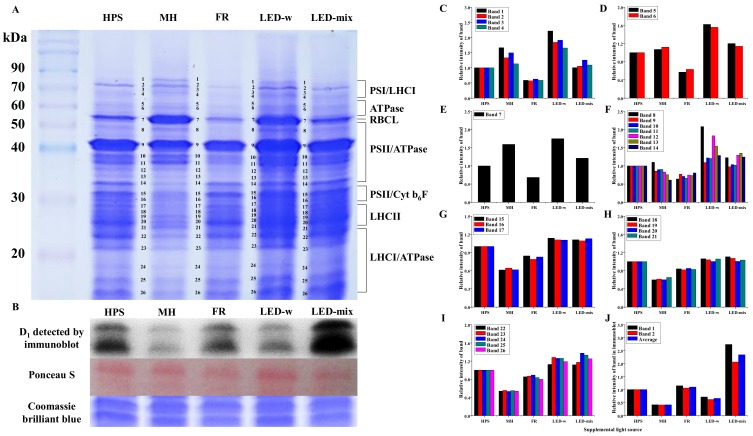
Chloroplast protein analysis (**A**) for *C. lanceolata* seedlings grown under supplementary light sources analyzed by sodium dodecyl sulfate polyacrylamide gel electrophoresis (SDS-PAGE), D_1_ protein of PSII detected by immunoblot and stained by Ponceau S or Coommassie brilliant blue (**B**), and the relative intensities of these protein bands (**C**–**J**) in SDS-PAGE gels or immunoblot membranes. PSI, Photosystem I complexes; PSII, Photosystem II complexes; LHCI, Light-harvesting complex I; LHCII, Light-harvesting complex II; ATPase, ATP synthase; RBCL, Rubisco large chain or ribulose bisphosphate carboxylase large chain; Cyt b_6_f, Cytochrome b_6_f; D_1_, a core protein in PSII, coded by *PsbA*.

About 26 apparent protein bands were found, with the mass ranging from 16 to 80 kDa (Figure 4A). We numbered these protein subunits according to their molecular masses (from the largest to the smallest). Additionally, seven putative protein groups were speculated according to the protein molecular mass described in Muneer [12,28,29] and Kügler [30]. The following description is based on the above hypothesis. The relative intensity of all putative protein groups, including PSI and LHCI subunits, LHCII, RBCL, ATPase, and PSII subunits was highly enhanced for seedlings in LED-w and LED-mix compared to that of seedlings in HPS and MH, and was reduced for seedlings in FR (Figure 4C–I).

The D_1_ protein of PSII, encoded by the *PsbA* genes, is an indispensable component of oxygenic photosynthesis. Results of an immunoblot analysis showed that the expression level of the D_1_ protein was dramatically enhanced in seedlings grown under LED-mix (Figure 4B). The intensity of the D_1_ protein in LED-mix-developed leaves was twice that of leaves in HPS and about 5 times that of leaves in MH (Figure 4J).

### 2.5. Analysis of H_2_O_2_ Localization and Antioxidant Enzyme Activities

**Figure 5:**
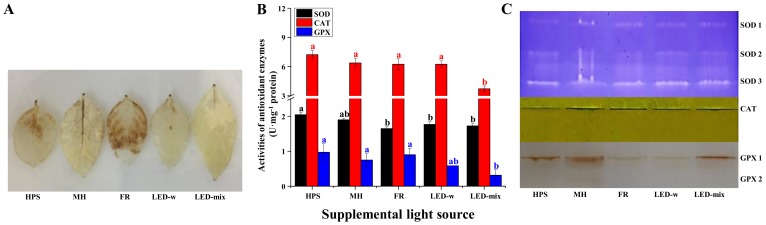
Localization of hydrogen peroxide (H_2_O_2_) in the leaves of *C. lanceolata* (**A**), the activities (**B**) and the expression analysis of antioxidant enzymes by native PAGE (**C**). Different small letters indicate the significant separation within treatments by Duncan’s multiple range test at a 0.05 level.

The hydrogen peroxide (H_2_O_2_) level was high in leaves of seedlings grown in FR and HPS, while it was significantly low in leaves of seedlings in LED-mix and LED-w (Figure 5A). The activity levels of sodium dismutase (SOD) in seedlings grown under LED-mix, LED-w, and FR were all lower than that of HPS. The activity of catalase (CAT) was significantly suppressed in seedlings developed in LED-mix compared to that of seedlings in the other four treatments. When compared to MH, LED-mix significantly inhibited, and FR largely increased, the guaiacol peroxidase (GPX) activity levels in seedlings (Figure 5B). The expression of these enzymes (Figure 5C) was slightly different from the enzyme activities described above. Three bands of SOD were found in native PAGE gels, and the abundance of all these SOD isoforms were similar. CAT had only one isoform, while two GPX isoforms were found. Supplementary lighting induced no significant differences in the CAT expression levels in seedlings, while GPX levels were lower for seedlings in FR, LED-w and LED-mix than for seedlings in HPS and MH.

### 2.6. Gene Expression Analysis

**Figure 6:**
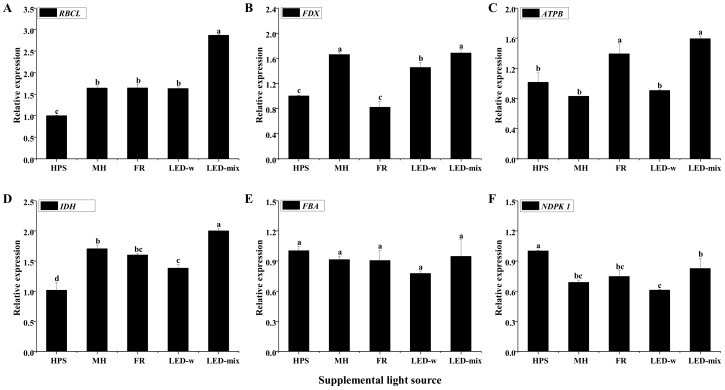
The relative gene expression of ribulose bisphosphate carboxylase large chain (RBCL) (**A**), ferredoxin (FDX) (**B**), ATP synthase subunit β (ATPB) (**C**), isocitrate dehydrogenase (IDH) (**D**), fructose-bisphosphate aldolase (FBA) (**E**), and nucleoside diphosphate kinase 1 (NDPK 1) (**F**). Different small letters indicate the significant separation within treatments by Duncan’s multiple range test at a 0.05 level.

The results of the quantitative real-time polymerase chain reaction (qRT-PCR) analysis indicated that *ribulose bisphosphate carboxylase large chain* (*RBCL*) was highly expressed in seedlings grown in LED-mix (Figure 6A), at a level almost 3 times that of seedlings in HPS and 1.74 times that of seedlings in MH. The expression of *ferredoxin *(*FDX*) was the highest for seedlings in LED-mix and MH, and the lowest for seedlings in FR (Figure 6B). The expression of *ATP synthase subunit β* (*ATPB*) was 56.8% and 92.0% higher for seedlings in LED-mix than that of seedlings in HPS or MH, respectively (Figure 6C). The expression of *isocitrate dehydrogenase* (*IDH*) was the highest for seedlings in LED-mix, 96.9% and 17.5% higher than that of seedlings in HPS or MH, respectively (Figure 6D). The expression of *fructose-bisphosphate aldolase *(*FBA*) in seedlings showed no significant differences among the treatments (Figure 6E). The expression of *nucleoside diphosphate kinase 1* (*NDPK 1*) was significantly lower for seedlings in LED-mix, LED-w, FR, and MH compared with that of seedlings in HPS (Figure 6F).

## 3. Discussion

Supplementary light has lately attracted increasing attention as it can enhance the yields, nutritional properties, and secondary metabolites of plants in greenhouses and plant factories. The spectrum analysis of the 5 supplementary light sources showed that the proportions of red light, blue light, and the sum of the 2 lights were highest for LED-mix, followed by LED-w. Photosynthetically active radiation (PAR, 400–700 nm) is the wavelength range that is effective for photosynthesis. LEDs are a promising PAR supplement in greenhouses and plant factories because its low energy consumption and the ability to supply discrete wavelengths can optimize seedling growth [31]. Plant development is also strongly influenced by the light spectrum [32]. The maximum action spectra for photosynthesis is in the blue and red ranges [33]. Combined red-blue LED light is proven to be effective in the production of many plant species, such as *Cucumis sativus*, *Lilium oriental*, *Fragaria × ananassa*, *Withania somnifera*, *Doritaenopsis*, *Lactuca sativa*, and *Gossypium hirsutum* [8,18,19,20,21,29,34]. We have also found that LED-mix is more efficient for biomass production, compact shoot development, and root growth improvement in *C. lanceolata* seedlings. Enhanced biomass and compact morphology with vigorous roots were also found in *Lactuca sativa* plants treated with LED-RBW lights [26]. It is encouraging that many studies report results that well support our findings. In addition to the comprehensive effects of different light sources, the analysis of blue and red light spectra takes on even more importance as it reveals further details on how they affect plant development. Red light significantly increases the stem length and leaf area of pea seedlings, while blue light largely enhances the seedling weight and chlorophyll level when compared to white light [35]. We found that the compactness of shoots grown under these 5 supplementary light sources was highly related to the blue light proportion. Namely, it was speculated that a higher blue light content resulted in more compact *C. lanceolata* seedlings. A high proportion of blue light from LEDs also resulted in more compact poinsettia, radish, soybean, and wheat [36,37]. Biomass distribution has been studied for several decades, but the explanation of plant biomass distribution is elusive. The present results showed that root growth was affected by the light spectrum. It was reported that root growth was enhanced by blue light and hindered by red light [21]. Plant metabolites including carbohydrates and phenol compounds are also largely regulated by the light spectrum [24,38,39]. In this study, starch and soluble sugar contents were highly enhanced in seedlings grown under LED-mix and largely reduced in FR-grown seedlings, which was highly in accordance with the biomass accumulation. Carbohydrate contents (starch, sucrose, glucose and fructose) were also significantly increased in *Doritaenopsis* and *Lactuca sativa* plants grown under combined red and blue LEDs compared to monochromatic red or blue LED and fluorescent light treatments [21,26]. Phenolic compounds function as antioxidants [40] and are important secondary metabolites in plants [41,42]. Numerous phenolic substances, including flavonoids and phenolic acids, have been found to be potentially disease-chemopreventive [43]. Our results showed that the total phenol and flavonoid contents were higher for seedlings grown under LED-w and LED-mix and lower for seedlings in FR-developed seedlings. Samuolienė et al. demonstrated that the phenolic concentration in lettuce green leaves grown under red light was increased by 52.7% [23]. However, supplemental red light had no influence on total phenols accumulation in spinach [44]. Other studies reported that UV light was more effective in optimizing the concentration of phenolic compounds [39,45,46]. In this study, the high concentrations of phenol and flavonoid for seedlings grown in LED-mix and LED-w are probably due to the enhancement of primary metabolism. Additionally, red light and blue light may also play a role in the accumulation of phenolic compounds. Therefore, LED-mix can be strategically used to enhance the biomass, nutritional value, medicinal properties, and stress resistance of *C. lanceolata* plants. The mechanisms responsible for such enhancements need further research.

Plants develop sophisticated mechanisms to adapt their structure and physiology to the light environment [47], especially in leaves. In this study, we found that the leaf number, specific leaf weight, and chlorophyll level of *C. lanceolata* were significantly increased for seedlings in LED-w and LED-mix, accounting for the enhancement of potential photosynthetic productivity for seedlings in LED-w and LED-mix. These changes were positively correlated with the proportion of blue light. The enhancement of *Eustoma* leaf thickness and chlorophyll with blue LED lighting supports our findings [10]. The stomata is a vital structure for photosynthesis. The stomatal size and density are indicators of plant adaptation and acclimation to environments [48]. The development of stomatal properties in tender leaves are largely affected by the light spectrum [11,49]. We found that the stomatal density, aperture, and conductance were highly enhanced in leaves developed in LED-mix, followed by those in LED-w, and were largely decreased for leaves in FR. The changes of the stomatal density were related to the proportion of red light, and the changes in the stomatal aperture were highly related to the proportion of blue light, as well as the summed proportion of blue and red lights. The stomatal density was affected by the light quality, but the roles of monochromatic lights remained controversial since different studies reported different results [10,11,49,50]. On the other hand, the effect of monochromatic lights on the stomatal aperture is relatively clear. It’s reported that blue light is more efficient in regulating the stomatal opening [11,49] and stomatal aperture is larger for plants under blue LEDs than for plants under red and white LEDs [10,51]. A weak blue light superimposed on red light induced a very rapid increase in the stomatal aperture in rice [52] and red light enhanced the blue light response of *Paphiopedilum* [53]. Our results implied that blue light effectively accelerated the stomatal opening, while red light maintains or enhanced the stomatal aperture and increases the stomatal density. A combination of red, blue, and white lights efficiently increased the stomatal conductance and the potential photosynthetic productivity by increasing the stomatal intensity and aperture. An increasing proportion of blue light also increased the stomatal conductance and the net photosynthetic rate in *Alternanthera brasiliana *[8]. The stomata provides plants with a dominant means to adjust to environmental changes [11]. Highly dense, small stomata is the best way to attain the highest stomatal conductance values at low atmospheric CO_2_ concentrations [54]. The risk of stomatal injury from mechanical damages or various stresses somewhat decreases with the increase of stomatal density. Thereby, stress resistance may be enhanced in plants that contained a large number of small stomata. The analysis of fossil records also showed that stomatal size decreased and stomatal density increased in plant leaves under CO_2_ impoverished atmospheres of the Permo-Carboniferous and Cenozoic glaciations [54]. Hence, plants grown under LED-mix may hold a higher stress resistance and maintain a high photosynthetic capacity in unfavorable environments.

The *FDX* gene, encoding a series of iron–sulfur proteins [55], occupies a key position both for the formation of NADPH and for mediating the cyclic electron flow around PSI [56,57,58]. The expression of *FDX* was the highest in seedlings developed in LED-mix, followed by those in LED-w, and was quite low for those in FR, following the proportion changes of blue light and the sum of red and blue lights. It somewhat implied that blue and red lights are efficient in regulating the expression of *FDX*. It is widely reported that the expression of photosynthetic genes and proteins is regulated by cryptochromes and phytochromes, which are largely affected by blue and red lights [12,59]. There are two related D_1_ and D_2_ proteins in the reaction center at the core of PSII [60]. They are involved in the light-driven primary and secondary electron transfer processes [61]. The dramatically higher expression level of the D_1_ protein detected by immunoblot in leaves developed in LED-mix indicated an enhanced photosynthetic potential. Besides this, the intensity trend of the D_1_ protein somewhat matched the proportion changes of red and blue lights of the 5 supplementary light sources. Namely, red and blue lights had a close relationship with the expression of the D_1_ protein. The expression of the D_1_ protein was also highly increased in carnations grown under red and blue LEDs [12]. Chloroplast protein analysis using SDS-PAGE represents a valuable tool to monitor the photosynthetic mechanisms [62]. PS I subunits, PSII subunits, LHCI subunits, LHCII subunits, RBCL, ATPase subunits, and Cyt b_6_f subunits were detected in *C. lanceolata* genotypes. Two photosystems (PSI and PSII) together with an ATPase and Cyt b_6_f complex enable the oxidation of H_2_O, NADP reduction, and ATP synthesis [63]. ATPase produces ATP at the expense of the proton motive force formed by light-driven electron-transfer reactions [64]. Cyt b_6_f complex mediates the electron transport between PSII and PSI and converts the redox energy into a part of the proton gradient used for ATP formation [65]. Ribulose-1,5-bisphosphate (RuBP) carboxylase/oxygenase (Rubisco) catalyzes the first step in the net photosynthetic CO_2_ assimilation and the photorespiratory carbon oxidation [66]. The intensity of all these protein subunits was largely enhanced for seedlings in LED-w and LED-mix, and was significantly decreased for seedlings in FR. Muneer reported that the expression level of these photosynthetic proteins was enhanced in carnations grown under red and blue lights [12]. Photosynthetic protein expression levels were higher in lettuce leaves grown under blue light than those grown under red or green lights [29]. Although there were some differences between the patterns of gene and protein expressions, the expression of *RBCL* and *ATPB* was all highly elevated in seedlings grown under LED-mix at both the gene transcription and translation levels. Rubisco biosynthesis and expression of *RBCL* were also increased in cucumber seedlings grown under blue and red lights [67]. It was concluded that LED-mix could enhance the expression of photosynthetic genes and proteins in *C. lanceolata* seedlings. Thereby, the development of photosystems was improved and the potential photosynthetic productivity was guaranteed in seedlings grown under LED-mix. In addition, two genes encoding carbon metabolism-related proteins were studied. *IDH *encodes an enzyme that catalyzes the oxidative decarboxylation of isocitrate, performing an essential role in the oxidative function of the tricarboxylic acid (TCA) cycle [68]. Proteins encoded by *FBA* plays a key role in glycolysis and gluconeogenesis [69]. TCA cycle and glycolysis are important components of respiration and carbon metabolism [70]. The expression of *IDH* was the highest for seedlings in LED-mix, whereas the expression of *FBA* in seedlings were not affected by the supplementary light treatments. FR, red and/or white lights upregulated several glycolytic genes of etiolated seedlings [7,71,72]. FR increased the level of NAD(H)-dependent enzymes by an unknown mechanism [73]. Taken together, light regulation of carbon metabolism-related genes is not evident [74].

A close relationship between plant stresses and reactive oxygen species (ROS) is commonly reported [12,28,75]. The localization of hydrogen peroxide showed that oxidative stresses were highly increased for seedlings in FR and HPS, while they were largely decreased for seedlings in LED-mix and LED-w. In the case of oxidative stresses, plants can control levels of ROS by activating the antioxidant enzyme activities as a defense mechanism [76,77,78]. In this study, it was found that the activities of antioxidant enzymes were correlated with oxidative stresses. An increase in the activities of antioxidant enzymes (SOD, GPX, and CAT) under oxidative stresses has been observed in several plants [79,80,81]. *Nucleoside diphosphate kinase 1* (*NDPK 1*) plays a major role in the synthesis of nucleoside triphosphates other than ATP. It also plays a role in the response to reactive oxygen species (ROS) stresses in *Arabidopsis *[82]. In accordance with the activity of SOD, the relative expression level of *NDPK 1* was significantly reduced in seedlings grown under LED-w, LED-mix, and FR compared with that of seedlings in HPS. The high radiant heat emissions and low photosynthetic active radiation (PAR) of HPS form an unfavorable environment and increase the thermal damage for plants [83]. The photosynthetic efficiency can also be drastically decreased by oxidative stresses [12]. We also found that the stomatal structure and properties, growth parameters, expression of photosynthetic genes and proteins, and the accumulation of photosynthates were all negatively influenced by oxidative stresses. Meanwhile, red and blue lights could alleviate the damage caused by some oxidative stresses [12]. Consistently, the oxidative stresses were quite low in *C. lanceolata* seedlings grown under LED-mix.

In this study, the effects of supplementary light sources and their monochromatic light proportions on plant growth and development were studied. Leaf and stomatal properties, chlorophyll, ROS, and antioxidant levels well explained the influence of supplementary light sources on photosynthesis from structural, physiological, and biochemical levels. In addition, the characterization of gene and protein expressions shed light on the molecular mechanisms of photosynthesis. However, a logical rigorous mechanism by which light sources affect photosynthesis has not been concluded from this study. To achieve this goal, further research of leaf anatomy, stomata and chloroplast structures, photosynthesis, and protein interactions should be carried out. LED technology enables the study of the effects of monochromatic lights. Studying the effects of monochromatic lights on the expression of specific genes and proteins will be the next step in research. Silencing and overexpression of genes can be used to study the functions of specific genes. Moreover, this study believes that the compactness of the shoots was closely related to blue light, and such mechanisms need further study.

## 4. Materials and Methods

### 4.1. Culture Conditions and Supplementary Light Treatments

This study chose HPS (BLV Licht-Und Vakuumtechnik, Steinhöring, Germany) and MH (SunLumen Lighting, Gyeongju, Republic of Korea) as the control because these two light sources are commonly used in commercial seedling production. An intersecting set of FR (Philips Lighting, Amsterdam, The Netherlands), LED-w (Victory Lighting, Seoul, Republic of Korea), and LED-mix (Custom made, SungKwang LED, Incheon, Republic of Korea) was used to figure out the optimal light source and their effects on the growth and physiology of *C. lanceolata*. Seedlings were cultured under various sources of supplementary light (Figure 1) in a glasshouse for 4 weeks, with a 16-h photoperiod with a 120 μmol·m^−2^·s^−1^ photosynthetic photon flux density (PPFD), in addition to an average daily maximum light intensity of 490 μmol·m^−2^·s^−1^ PPFD coming from the sun with a 14-h photoperiod, 30 °C/22 °C day/night temperatures, and 70% relative humidity. The spectrum of light sources was measured by spectroradiometers (International Light Technologies, Peabody, MA, USA). The growth parameters were recorded and plants were harvested for physiological and biochemical analyses after four weeks of treatment.

### 4.2. Scanning Electron Microscopic (SEM) Analysis of the Stomata

Leaves were carefully excised and kept in 1.5 mL centrifuge tubes where 2.5% glutaraldehyde was added, followed by an 8-h incubation at 4 °C. The glutaraldehyde was washed using a 0.1 M phosphate buffer (PBS, pH 7.0). Samples were fixed in 1% osmic acid (OsO_4_) for 2 h at 4 °C. Subsequently, they were dehydrated in graded series of ethanol (30%, 50%, 70%, 90%, and 100%). After dehydration, samples were dried, gold coated, examined, and photographed under DS-130 ISI (Oxford, UK) scanning electron microscope.

The stomatal properties were analyzed using ImagJ software (version 1.8.0; available online: https://imagej.nih.gov/ij/download.html). The guard cell length (stomatal length), width of guard cell pair (stomatal width), stomatal pore length, and pore width were measured according to the definition of Sack et al. [48] and Savvides et al. [51]. The stomatal size was calculated as the guard cell length multiplied by the width of the guard cell pair [54,84]. The stomatal density (the number of stomata per area) was calculated as the stomata number divided by the area where the stomata number was recorded [48,54]. The stomatal aperture was calculated as the stomatal pore width divided by the stomatal pore length [85]. The stomatal conductance was measured by a SC-1 leaf porometer (Meter, Pullman, WA, USA) according to the manual.

### 4.3. Chlorophyll, Carbohydrate, and Soluble Protein Analyses

The chlorophyll level was measured by Plus Chlorophyll Meter (Spectrum technologies, Wales, UK). The contents of starch and soluble sugars were measured by the Anthrone colorimetric method according to Ren et al. [86]. Soluble proteins were extracted with a sodium phosphate buffer and measured colorimetrically using a Bradford Reagent (Sigma-Aldrich, St. Louis, MO, USA) according to the manual.

### 4.4. Total Phenol and Flavonoid Analyses

Secondary metabolites were extracted with 80% methanol. The contents of total phenol and flavonoid were estimated according to the method outlined by Manivannan et al. [87].

### 4.5. Localization of Hydrogen Peroxide (H_2_O_2_)

Leaves were immersed completely in 0.1% 3,3′-Diaminobenzidine (DAB), followed by a 15-minute vacuum infiltration and a 2-h incubation in darkness. Subsequently, leaves were washed three times with distilled water and immersed in 100% ethanol. DAB reactions were examined in leaves after 10 min of inoculation in boiling ethanol.

### 4.6. Activities of Antioxidant Enzymes and Native Polyacrylamide Gel Electrophoresis (Native-PAGE) Analysis

The activities of SOD, CAT, and GPX were estimated based on the protocol of Muneer et al. [75]. Native-PAGE analysis of the antioxidant enzymes and the development of enzyme isomers were performed according to Soundararajan et al. [88].

### 4.7. Sodium Dodecyl Sulfate Polyacrylamide Gel Electrophoresis (SDS-PAGE) Analysis and Immunoblot Assay

Chloroplast proteins were extracted by the method of Muneer et al. [12,29] and separated by sodium dodecyl sulfate polyacrylamide gel electrophoresis (SDS-PAGE). Protein bands in the gels were analyzed according to the method of Muneer et al. [12] and the intensity of these bands were calculated using ImagJ software (version 1.8.0). Total proteins were extracted using a Tris-HCl buffer (100 mM Tris-HCl with a pH of 7.8, 1 mM EDTA-Na2, 2% PVP, 1% Triton X-100, and 0.07% β-mercaptoethnol). Extracted proteins (25 µg) were mixed with a loading buffer (240 mM Tris-HCl (pH 6.8), 40% glycerol, 8% SDS, 0.04% bromophenol blue, and 5% β-mercaptoethanol) and separated by SDS-PAGE. The expression of the D_1_ protein (anti-PsbA) was analyzed by immunoblotting as described by Shen et al. [89].

### 4.8. Primer Design and qRT-PCR

Primers were designed using Premier 5.0 (Premier Biosoft Inc., Palo Alto, CA, USA). Total RNA was extracted from seeds using a total RNA extraction kit (Real Biotech Corporation, Taiwan, China) according to the manufacturer’s protocol. First-strand cDNA was synthesized using a GoScript™ reverse transcription system (Promega, Fitchburg, WI, USA), and qRT-PCR was performed based on the method of Wang et al. [90]. Relative expression levels were calculated using 2^−ΔΔ*C*t^ method [91]. The *C. lanceolata* actin gene was used as the control to normalize the qPCR results with no significant changes in the transcriptional levels at any of the supplementary light conditions, and other primers used were listed in Table 4. All qRT-PCR reactions were performed in triplicate with at least three biological replicates.

### 4.9. Statistical Analysis

The experimental assays used to obtain all results were repeated at least three times and are presented as the mean ± standard error (SE). Data collected were subjected to analysis of variance (ANOVA) followed by Duncan’s multiple range test at *p* < 0.05 to find the statistical significance among the treatments by the SAS (Statistical Analysis System, V. 6.12, Cary, NC, USA) program.

## 5. Conclusions

This study demonstrated that the spectrum of supplementary light sources is actively involved in the various growth and development processes of seedlings. Plants adapted their structures, the expression of genes and proteins, and various metabolic processes to the light environment. The expression of photosynthetic genes and proteins in seedlings was boosted by LED-mix. Red and blue lights played a vital role in the expression of the D_1_ protein (PS II) and accelerated the formation of ferredoxin-like iron-sulfur cluster proteins (PSI). Furthermore, red and blue lights could alleviate the damages caused by oxidative stresses. Taken altogether, LED-mix could enhance the photosynthetic potential productivity by making full use of the light energy and alleviating the damage caused by oxidative stresses, resulting in a higher biomass, compact shoots, improved roots, and a larger accumulation of starch, soluble sugars, total phenol, and flavonoids. Consequently, LED-mix is recommended to be used in the production of high-quality *C. lanceolata* seedlings, which have a high nutritional value and elevated medicinal properties.

## Figures and Tables

**Table 1:** The irradiance distribution of the supplementary light sources.

Supplementary Light Source	Irradiance (μW·cm^−2^)								
	UV (250−380 nm)	Violet (381–450 nm)	Blue (451–495 nm)	Green (496–570 nm)	Yellow (571–590 nm)	Orange (591–620 nm)	Red (621–710 nm)	FR (711–850 nm)	IR (851–1050 nm)
HPS	11.0	46.8	54.9	356.0	509.5	906.1	434.9	655.7	25.2
MH	118.5	703.2	314.1	788.9	296.6	258.6	311.6	147.5	61.0
FR	1.9	0.7	0.2	0.5	0.5	4.5	191.6	934.5	1865.6
LED-w	12.3	329.2	668.7	917.8	268.2	345.5	385.2	33.4	39.8
LED-mix	8.5	445.6	874.7	258.0	69.7	93.6	1235.6	8.8	5.6

**Table 2:** Guard cell length, width of guard cell pair, and stomatal pore length and width in leaves of *C. lanceolata* seedlings as affected by supplementary light sources. Different alphabets indicate the significant separation within columns by Duncan’s multiple range test at a 0.05 level.

Supplementary Light Source	Guard Cell Length (µm)	Width of Guard Cell Pair (µm)	Pore Length (µm)	Pore Width (µm)
HPS	16.9 ± 0.4 ^a^	7.9 ± 0.3 ^b^	11.2 ± 0.3 ^a^	3.3 ± 0.3 ^a^
MH	16.0 ± 0.4 ^a^	7.9 ± 0.4 ^b^	8.9 ± 0.3 ^b^	3.4 ± 0.3 ^a^
FR	15.8 ± 0.6 ^a^	8.9 ± 0.4 ^a^	7.8 ± 0.3 ^cd^	1.8 ± 0.2 ^b^
LED-w	15.7 ± 0.4 ^a^	8.7 ± 0.4 ^ab^	7.6 ± 0.3 ^d^	2.4 ± 0.2 ^b^
LED-mix	15.6 ± 0.4 ^a^	7.8 ± 0.3 ^b^	7.9 ± 0.3 ^c^	4.0 ± 0.2 ^a^

**Table 3:** The physiological parameters of *C. lanceolata* seedlings as affected by supplementary light sources. Different alphabets indicate the significant separation within columns by Duncan’s multiple range test at a 0.05 level.

Supplementary Light Source	Soluble Sugar Content (% of Fresh Mass)	Starch Content (% of Fresh Mass)	Protein Content (% of Fresh Mass)	Total Phenol Content (µg·g^−1^ Fresh Mass)	Total Flavonoid Content (µg·g^−1^ Fresh Mass)
HPS	2.66 ± 0.08 ^b^	1.51 ± 0.06 ^ab^	0.55 ± 0.03 ^b^	0.53 ± 0.03^ b^	0.65 ± 0.04^ b^
MH	2.51 ± 0.08 ^b^	1.35 ± 0.03 ^b^	0.71 ± 0.01 ^a^	0.30 ± 0.02^ c^	0.37 ± 0.02^ c^
FR	1.69 ± 0.15 ^c^	1.15 ± 0.05 ^c^	0.74 ± 0.03 ^a^	0.28 ± 0.02^ c^	0.35 ± 0.02^ c^
LED-w	1.52 ± 0.10 ^c^	1.50 ± 0.07 ^ab^	0.68 ± 0.04 ^a^	1.10 ± 0.06^ a^	1.35 ± 0.07^ a^
LED-mix	3.28 ± 0.11 ^a^	1.62 ± 0.01 ^a^	0.70 ± 0.01 ^a^	0.97 ± 0.02^ a^	1.18 ± 0.02^ a^

**Table 4:** The nucleotide sequences of the PCR primers used to assay the gene expression by quantitative real-time PCR.

Gene	Forward Primers	Reverse Primers
*β-Actin*	5′-CGAGAAGAGCTACGAGCTACCCGATGG-3′	5′-CTCGGTGCTAGGGCAGTGATCTCTTTGCT-3′
*ATPB*	5′-TGCCTTCTGCTGTGGGTTAT-3′	5′-GGTCGGTCAAATCGTCTGC-3′
*FDX*	5′-CTTCGGCGTTTCTTCGT-3′	5′-CTGCCAAACCCTTGATAACT-3′
*RBCL*	5′-GCTTACCCATTAGACCTTT-3′	5′-GGGACGACCATACTTGTT-3′
*FBA*	5′-ACAGGTGGGCTCTTC GTG-3′	5′-CCTTGGGTGGTGGTTTCA-3′
*IDH*	5′-TGACGGAGGTTATGTATGG-3′	5′-AATGCTGTTCGTGCTGGT-3′
*NDPK 1*	5′-AGAGGCTTGGTTGGTGAGA-3′	5′-AGAGGCAGCAGGGTTTGT-3′
